# The Red Shift in Estrogen Research: An Estrogen-Receptor Targeted *aza*-BODIPY–Estradiol Fluorescent Conjugate

**DOI:** 10.3390/ijms26157075

**Published:** 2025-07-23

**Authors:** Tamás Hlogyik, Noémi Bózsity, Rita Börzsei, Benjámin Kovács, Péter Labos, Csaba Hetényi, Mónika Kiricsi, Ildikó Huliák, Zoltán Kele, Miklós Poór, János Erostyák, Attila Hunyadi, István Zupkó, Erzsébet Mernyák

**Affiliations:** 1 Institute of Pharmacognosy, University of Szeged, Eötvös u. 6, H-6720 Szeged, Hungary; tamas.hlogyik@gmail.com (T.H.); hunyadi.attila@szte.hu (A.H.); 2 Institute of Pharmacodynamics and Biopharmacy, University of Szeged, Eötvös u. 6, H-6720 Szeged, Hungary; bozsity-farago.noemi@szte.hu (N.B.);; 3 Department of Pharmacology and Pharmacotherapy, Medical School, University of Pécs, Szigeti út 12, H-7624 Pécs, Hungary; 4 Department of Biochemistry and Molecular Biology, University of Szeged, Közép fasor 52, H-6726 Szeged, Hungary; 5 Department of Medicinal Chemistry, University of Szeged, Dóm tér 8, H-6720 Szeged, Hungary; 6 Department of Laboratory Medicine, Medical School, University of Pécs, Ifjúság útja 13, H-7624 Pécs, Hungary; poor.miklos@pte.hu; 7 Molecular Medicine Research Group, János Szentágothai Research Centre, University of Pécs, Ifjúság útja 20, H-7624 Pécs, Hungary; 8 Department of Experimental Physics, Faculty of Sciences, University of Pécs, Ifjúság útja 6, H-7624 Pécs, Hungary; 9 Molecular Biophysics Research Group, János Szentágothai Research Centre, University of Pécs, Ifjúság útja 20, H-7624 Pécs, Hungary; 10 HUN-REN-SZTE Biologically Active Natural Products Research Group, Eötvös u. 6, H-6720 Szeged, Hungary

**Keywords:** estradiol, fluorescent labeling, *aza*-BODIPY, red-emission, CuAAC reaction, luciferase, docking

## Abstract

Estradiol (E2) plays an important role in cell proliferation and certain brain functions. To reveal its mechanism of action, its detectability is essential. Only a few fluorescent-labeled hormonally active E2s exist in the literature, and their mechanism of action usually remains unclear. It would be of particular interest to develop novel labeled estradiol derivatives with retained biological activity and improved optical properties. Due to their superior optical characteristics, *aza*-BODIPY dyes are frequently used labeling agents in biomedical applications. E2 was labeled with the *aza*-BODIPY dye at its phenolic hydroxy function via an alkyl linker and a triazole coupling moiety. The estrogenic activity of the newly synthesized fluorescent conjugate was evaluated via transcriptional luciferase assay. Docking calculations were performed for the classical and alternative binding sites (CBS and ABS) of human estrogen receptor α. The terminal alkyne function was introduced into the tetraphenyl *aza*-BODIPY core via selective formylation, oxidation, and subsequent amidation with propargyl amine. The conjugation was achieved via Cu(I)-catalyzed azide–alkyne click reaction of the *aza*-BODIPY-alkyne with the 3-O-(4-azidobut-1-yl) derivative of E2. The labeled estrogen induced a dose-dependent transcriptional activity of human estrogen receptor α with a submicromolar EC_50_ value. Docking calculations revealed that the steroid part has a perfect overlap with E2 in ABS. In CBS, however, a head-tail binding deviation was observed. A facile, fluorescent labeling methodology has been elaborated for the development of a novel red-emitting E2 conjugate with substantial estrogenic activity. Docking experiments uncovered the binding mode of the conjugate in both ABS and CBS.

## 1. Introduction

**Figure 1:**
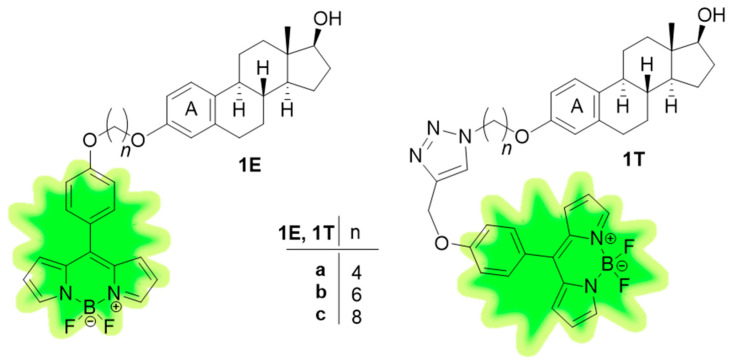
Structures of estrogenic BODIPY-labeled E2 conjugates (**1E**, **1T**).

Estradiol (E2), as the main form of estrogen, plays an important role in hormone-dependent diseases by stimulating cell proliferation. Nevertheless, its biological activity is not limited to the hormonal system. For example, it plays a crucial role in skeletal muscle homeostasis [1] and can modulate essential brain functions [2]. The high biological significance of E2 justifies its adequate detectability. Gajadeera et al. reviewed fluorescent ligands for estrogen receptor (ER) and discussed the basic structure-activity relationships [3]. Of the synthetically accessible positions, substitutions at C-6(α/β), C-7(β), C-11(β), C-16(α/β), and C-17(α) with certain functional groups are well-tolerated by estrogen receptor (ER) without significant decreases in the binding affinity. A large body of literature suggests that modifications at the A-ring are detrimental. Nevertheless, etherification of the phenolic hydroxy function of E2 and attachment of a fluorescent label via shorter or longer linkers might result in labeled compounds with retained estrogenic behavior [4,5]. However, the exact mechanism of action of these labeled estrogens often remains unclear. We have recently published the labeling strategy of E2 at its 3-OH group via alkyl linkers. Green fluorescent 4, 4-difluoro-4-bora-3a,4a-diaza-s-indacene (BODIPY) dyes have been attached as fluorophores. The labels were connected to the steroid via three different freely rotating linkers through an ether or a triazole coupling moiety, furnishing conjugates **1E** or **1T**, respectively (Figure 1) [6]. Results of the docking calculations on the human estrogen receptor alpha (hERα) suggested that the four-carbon linker containing **1Ea** and **1Ta** have the best calculated per-atom binding affinities (efficiency indices, EIs). Compound **1Ta** formed an interaction with H524 via its 17-OH function in classical binding site (CBS), which is considered to be essential for the proper binding and hormonal activity. All labeled compounds exerted potent estrogenic action in a cell-based assay in MCF-7 (ER-positive) breast cancer cell line. The most potent compound (**1Ea**) stimulated considerably the ER transcriptional activity and downstream signaling. The direct interaction of **1Ea** with recombinant ERα was confirmed by the competitive radioligand binding assay. Based on the above results, candidate conjugates with a four-carbon alkyl linker at C-3-O (**1Ea** and **1Ta**) represent a promising basis for further research.

BODIPY derivatives are one of the most frequently used fluorophores [7]. This is due to their excellent chemical and biological stability, easy availability, and wide range of optical applications [8]. The latter might be limited by the relatively low absorption and emission maxima, which, however, can be shifted towards higher wavelengths by controlled chemical transformations. The two most desired colors from the electromagnetic spectrum are green and red, which have found extensive utilization in several fields [9]. Biomedical imaging usually requires multicolor options in order to perform colocalization but suppress cellular autofluorescence in microscopic investigations [10,11]. The red or infrared counterparts of BODIPY dyes contain a nitrogen atom in the *meso* position and they are called *aza*-BODIPY derivatives [12]. They came into focus of attention due to their superior optical characteristics. Beside their physiological, chemical, and thermal stability, their high molar extinction coefficients and their red or infrared emission make them versatile tools in biological applications. These include, but are not limited to fluorescence bioimaging, phototherapy, and several combined therapies [13]. Red-emitting dyes are particularly exploitable for imaging living tissues and cells, owing to their deep tissue penetration and ability to avoid visible light absorption. They cause less light damage than their green emitting counterparts. The red signal is highly distinguishable from the tissue autofluorescence. An additional advantage is that the optical setup is also much simpler when investigating in the red range. Bioimaging techniques with high spatial and temporal resolution facilitate the specialized application of specific red-emitting dyes.

Certain E2-BODIPY conjugates, labeled mainly at position 17α, are described in the literature [14,15]. Nevertheless, only a few E2-*aza*-BODIPY conjugates were synthesized [16]. Labeling was performed at position 17α via Sonogashira coupling of 17α-ethynylestradiol and the iodophenyl counterpart of tetraphenyl *aza*-BODIPY. Estrogenic effects of the latter conjugates have not been investigated. An explanation for the lower number of *aza*-BODIPY estrogen derivatives in the literature may be the later development and less efficient synthesis of the *aza*-BODIPY scaffold.

Literature reveals three main synthetic strategies for the construction of the *aza*-BODIPY core. These include O’Shea’s method, leading to symmetrical dyes relies on chalcone synthesis, followed by Michael-addition with nitromethane, cyclization using an ammonium salt, and complexation with boron trifluoride [17]. This simple and mild strategy allows the formation of the *aza*-BODIPY in low yields. The least efficient step in the reaction sequence is cyclization. Ammonium formate or ammonium acetate are the most commonly used ammonia sources, applied under solventless conditions or in alcohol solvents. The best yields, however, fall into the range of 40–50% [12]. Jameson et al. reported a microwave-assisted synthesis of the aza-bridge using ammonium acetate as a reagent in trifluoroethanol as solvent at 70 °C in 47% yield [18]. Comparable effectiveness was achieved by Gut et al. in acetic acid solvent using urea as a reagent in a microwave reactor at 140 °C [19]. Improving the efficiency of this reaction step would be a major breakthrough in the synthesis of these valuable fluorophores. O’Shea et al. created the non-symmetrical *aza*-BODIPY core via the condensation of a nitrosopyrrole and a pyrrole as the key step. This process requires the introduction of a nitrosation step as well. Note, however, that Carreira’s method avoids this reaction step [20]. It is based on the direct cyclization of a 2,3,4- and a 2, 4-substituted pyrrole under the conditions of nitrosation and subsequent complexation with boron trifluoride. The yield of this method is somewhat higher. The third, but least efficient, procedure belongs to Lukyanets et al., which is based on the Grignard reaction of phthalonitrile with aryl magnesium bromides [21].

**Figure 2:**
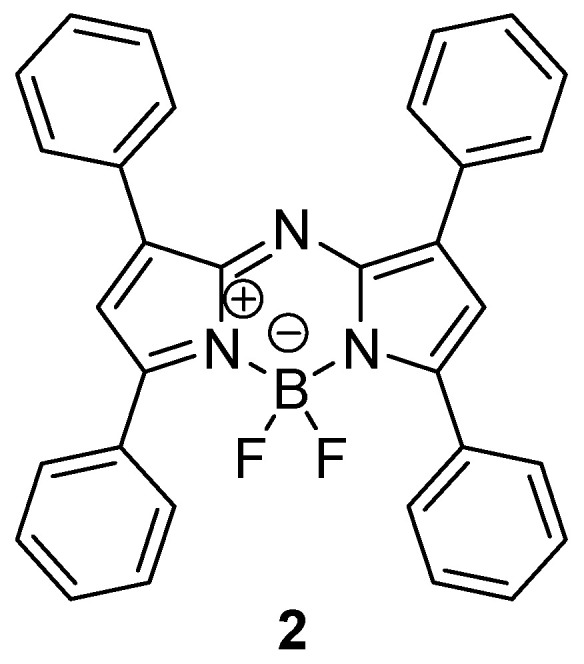
The structure of the tetraphenyl *aza*-BODIPY **2**.

We have recently published the reaction route leading to tetraphenyl *aza*-BODIPY **2** and some of its derivatives (Figure 2) [22]. The low micromolar in vitro photodynamic activity of compound **2** was confirmed on the epidermoid carcinoma cell line A431. The negligible dark, but potent, phototoxicity makes dye **2** promising for other biomedical applications.

Based on our recent results obtained for BODIPY–E2 conjugates [6], here we aimed to synthesize an *aza*-BODIPY–E2 compound via Cu(I)-catalyzed azide–alkyne click reaction (CuAAC) strategy. After the selective introduction of one terminal alkyne function to dye **2**, the fluorescent labeling was planned starting from the *aza*-BODIPY-alkyne and the steroid azide via inserting a C4-long alkyl linker. The investigation of the estrogenic activity of the newly synthesized conjugate was intended in a luciferase transcriptional assay. The binding mode and affinity of the labeled E2 was determined by computational simulations.

The red-emitting fluorescent conjugate, which retains estrogenic activity, may serve as a valuable tool for studying the uptake and transport pathways characteristic of estrogens, as well as the biotransformation processes of estrogen molecules themselves, with particular emphasis on the role of estrogen-binding proteins. Owing to its favorable photophysical characteristics, it may also facilitate the identification of novel biological effects associated with estrogens.

## 2. Results and Discussion

### 2.1. Chemistry

Our first priority was the synthesis of a tetraphenyl *aza*-BODIPY derivative, which might effectively be connected to E2. Dye **2** (Figure 2) has no functional groups with the ability of conjugation in its intact form. The selective incorporation of a single appropriate group required careful consideration. Postfunctionalization of the hetero- or carbocyclic aromatic rings allows the introduction of certain groups, but the challenge here lies in a selective modification, i.e., the incorporation of only a single desired function. Halogenation of the pyrrole rings might result in widely transformable compounds, but monosubstitution at only a single pyrrole moiety is difficult to achieve [23]. A more favorable outcome may be the Vilsmeier–Haack formylation of the *aza*-BODIPY core. Literature suggests that it occurs selectively on one of the pyrrole rings, double substitution does not take place, and the other aromatic rings remain intact [24]. Oxidation of the formyl group allows the formation of the carboxylic acid counterpart, which might be readily transformed into a terminal alkyne using propargyl amine as the reagent. This strategy provides a key alkyne intermediate, which can be effectively coupled to an azide in a CuAAC reaction. In view of the above, we chose a formylation–oxidation–amidation strategy.

**Scheme 1:**
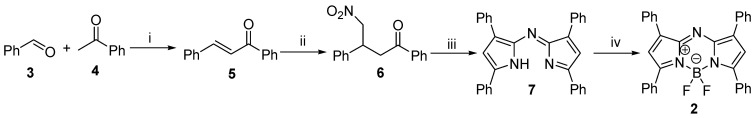
Reagents and conditions: (i) NaOH (2 equiv), methanol, rt, 2 h; (ii) catalytic amount of NaOH, methanol, nitromethane (5 equiv), 60 °C, 3 h; (iii) NH_4_OAc (20 equiv), 100 °C, 3 min, microwave irradiation (MW); (iv) Et_3_N (10 equiv), toluene, rt, 15 min, BF_3_.OEt_2_ (15 equiv), reflux, 5 h.

The synthesis of dye **2** was performed as reported earlier [22], but with a special focus on increasing the efficiency of the reaction sequence (Scheme 1). The condensation reaction of benzaldehyde **3** and acetophenone **4** resulted in chalcone **5**, which was subjected to Michael addition using nitromethane as reagent. These two steps have not been modified, but the dimerization of nitromethylene derivative **6** was investigated under different reaction conditions in a microwave reactor (Table 1). The methodologies used here were planned according to the previously published results and the principles of green chemistry. Two different reagents and two solvents have been applied. Although ethanol is considered to be a green solvent [25], the use of water as a reaction medium was also tested. In addition, the dimerization was investigated under solvent-free conditions as well. With ammonium acetate, more efficient conversion was achieved in ethanol than in aqueous media, but solvent-free heating at 100 °C for 3 min proved to be the most optimal choice (Entry 5). At higher temperatures, significant decomposition of starting material **6** was observed (Entry 6). As entries 7–16 indicate, an acid additive was required when using urea as the ammonia source. Two natural products, namely acetic acid (AA) (formerly used as solvent by Gut et. al. [19]) and citric acid (CA), have been tested. CA is a nontoxic, biocompatible, readily available compound, which has found its utilization in several fields of bioorganic chemistry [26]. Urea allowed the reaction to be carried out efficiently in aqueous media (Entries 11, 12 and 15, 16). Urea is not only famous for being the first organic compound produced by a chemist, but it is also one of the most common organic metabolites. Other advantageous properties are its nontoxic and water-soluble nature [27]. Considering the data in Table 1, two results are worth highlighting. Entries 5 and 16 reveal the two conditions, which led to the highest yields. Entry 16 describes the greenest efficient procedure.

**Scheme 2:**
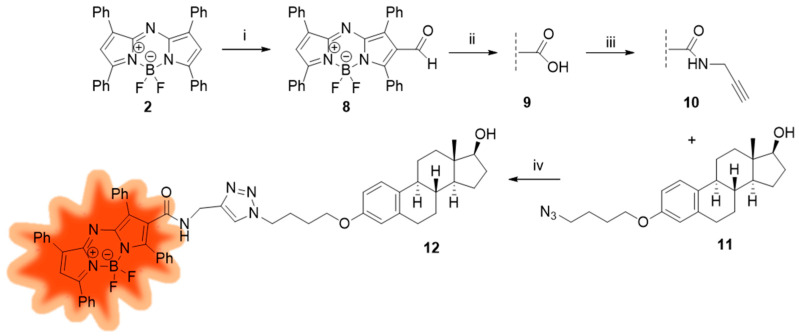
Reagents and conditions: (i) POCl_3_, DMF 0 °C to rt, 30 min; DCE, 70 °C, 24 h; NaHCO_3_, 0 °C to rt, 1 h; (ii) NaClO_2_, NH_2_SO_3_H, THF/H_2_O (3/1), rt, 30 min; (iii) EDC, HOBt, propargylamine, CH_2_Cl_2_, rt, 4 h; (iv) CuI, SPhos, DIPEA, toluene, reflux, 2 h.

The transformation of tetraphenyl *aza*-BODIPY **2** to its terminal alkyne counterpart was carried out via selective postfunctionalization, starting with formylation at C-2 (Scheme 2). Only mono-substitution took place in Vilsmeier–Haack formylation, resulting in carbaldehyde derivative **8**, which underwent oxidation to its carboxylic acid counterpart **9**. We chose a mild method from the literature described for the selective oxidation of a formyl group on the BODIPY core [24]. Pinnick oxidation was achieved by using sodium chlorite as the oxidizing agent and sulfamic acid (SA) as an additive in a THF/H_2_O = 3/1 mixture [28]. SA is an efficient and green heterogeneous catalyst utilized in diverse areas of organic synthesis [29]. This mild and effective oxidation method resulted in the formation of the desired carboxylic acid **9** in high yield. No side reactions occurred. To the best of our knowledge, this oxidation method has so far been applied only to BODIPY derivatives [24] and not to *aza*-BODIPY compounds. Next, we focused on the synthesis of an amide using propargyl amine as the reagent. The activation of the carboxylic acid was achieved with N-ethyl-N′-(3-dimethylaminopropyl)carbodiimide (EDC). 1-Hydroxybenzotriazole (HOBt) was used as an additive. The desired terminal alkyne **10** was obtained in high yield. Finally, fluorescent labeling of E2 was performed via CuAAC reaction of dye **10** as a terminal alkyne and E2-azide **11**. Azide **11** was selected on the basis of our previous work showing that conjugates bearing four-carbon linkers performed the best [6]. Conjugation of the dye to E2 was carried out via a slight modification of our previously applied click methodology. Compounds **10** and **11** served as key intermediates in the CuAAC reaction, while CuI was used as a catalyst, 2-dicyclohexylphosphino-2′,6′-dimethoxybiphenyl (SPhos) as an accelerating ligand, and *N*,*N*-diisopropylethylamine (DIPEA) as a base. The use of SPhos instead of triphenylphosphine (PPh_3_) was justified by the fact that the oxide derivative formed from the latter accompanied the target compound during column chromatographic purification. To avoid the formation of triphenylphosphine oxide (PPh_3_O), another phosphine-based ligand was chosen, which, due to the presence of biphenyl and cyclohexyl substituents, falls in a different polarity range and thus it does not contaminate the target compound during purification.

The structures of the newly synthesized *aza*-BODIPY derivatives **9**, **10** and the *aza*-BODIPY-E2 conjugate **12** were evaluated from ^1^H and ^13^C NMR spectra (Supplementary Material, copies of ^1^H and ^13^C NMR spectra). In assigning the signals, our primary focus was to highlight the changes indicative of the success of each reaction step. A similar approach was adopted in our recent two publications [6,22]. The following section outlines the main observations and interpretations based on the proton NMR spectra.

The presence of the carboxyl group in compound **9** (Scheme 2) is indicated by a singlet appearing at approximately 12.88 ppm in the ^1^H NMR spectrum. The formation of the amide bond with propargylamine can be inferred from the appearance of a singlet around 2 ppm, corresponding to the acetylenic proton, a multiplet near 4 ppm assigned to the NCH_2_ group, and a triplet around 5.5 ppm corresponding to the NH proton. In the spectrum of the triazole-containing conjugate **12**, signals in the 3.9–4.5 ppm range are attributed to the OCH_2_ group and the two NCH_2_ groups. Furthermore, the spectrum also displays characteristic signals corresponding to the *aza*-BODIPY and steroid moieties. Taken together, these observations suggest the formation of the desired conjugate.

### 2.2. Pharmacology

**Figure 3:**
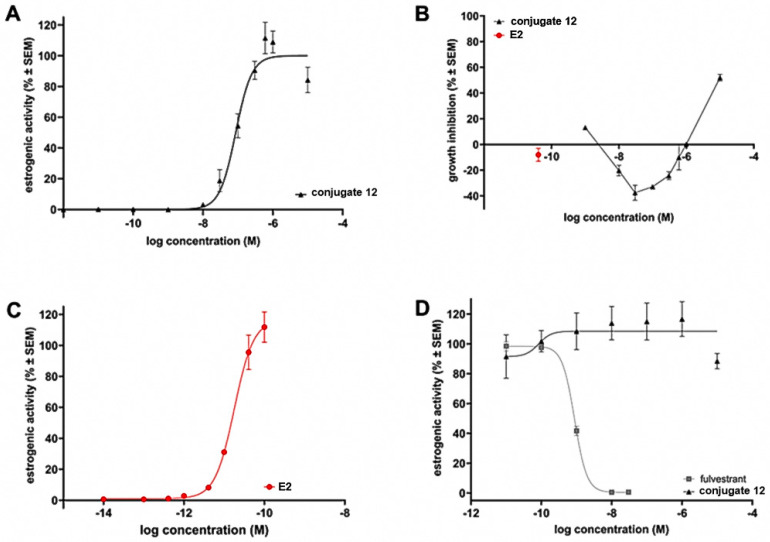
(**A**) The effect of *aza*-BODIPY–E2 conjugate **12** on the ER-mediated transcription in the luciferase reporter model. (**B**) Growth inhibition of conjugate **12** and E2, measured by MTT assay. (**C**) Estrogenic activity of reference E2 in the luciferase reporter model. (**D**) Antiestrogenic effect of conjugate **12** and fulvestrant (in the presence of E2) as reference.

The estrogenic activity of *aza*-BODIPY–E2 conjugate **12** was measured on the T-47D-KBluc cell line [30]. Since ER-R is a nuclear receptor, in response to the estrogen signal, receptors dimerize and enter the nucleus to bind to DNA. In the nucleus, the estrogen-ER-R complex binds into a specific region in the DNA, referring to estrogen-responsive elements (ERE), and exerting its gene-regulating effect. The used cell line is stably transfected with a triplet ERE-promoter-luciferase reporter gene construct, which results in a high sensitivity to estrogens and may prove estrogen activity [31]. When the ligand activates the ER in the T-47-D-KBluc cells, the complex binds to the ERE in the nucleus, and in addition to the transcription of the regular target regions, luciferase enzyme genes were also expressed, resulting in active luciferase enzyme in the cytoplasm. Therefore, it can indicate the estrogen response into our new labeled estrogen. In our studies, labeled compound **12** significantly elevated the bioluminescence signal caused by luciferin after 24 h of treatment. It was concentration-dependent with the 86 nM IC_50_ value (Figure 3A). Compound **12** exerted its maximal effect at 0.3 µM (300 nm), while a slight decline (down to 88%) was observed after a high concentration (30 µM). We hypothesize that the direct cytotoxic effect of compound **12** may be responsible for this decline. The MTT assay was performed at the same conditions as the luciferase assay to prove that theory. We can see, in the antiproliferative MTT results, that under the same estrogen-depleted conditions, the strong estrogenic activity of compound **12** results in slight cell number elevation in the 1 nM and 10 µM test concentration range (in parallel with the high estrogenic activity). In turn, at a concentration of 30 µM, where around 50% inhibition can be seen, the estrogenic activity slowly decreased (Figure 3B). The same experiment was performed in the presence of 40 pM 17β-estradiol to ensure that this effect is not due to an antiestrogen effect. The reference agent, fulvestrant, exerted the expected effect, while compound **12** did not change the maximal estrogenic effect caused by E2 significantly, not even in the high concentrations.

Growth inhibition after 72 h caused by derivative **12** has also been investigated in breast cancer cell lines with different receptor patterns (Table 2). Results show that the compound has a moderate antiproliferative effect in the triple-negative MD-MBA-231 breast cell line, which is not dose-dependent and remains at the same position at the examined concentration rate (0.1–30 µM). In the ER + T-47D cells, the compound did not cause any growth inhibition below the 30 µM test concentration. This is similar to the effect on the ER+ overexpressed and transfected T-47D-KBluc cells described previously, where proliferation induction can be found after 24 h in the concentration range of 0.01–1.0 µM. It is consistent with the luciferase assay results, where intensive estrogenic activity can be found at the same concentration range. The compound does not affect the growth of the intact NIH/3T3 cells.

**Figure 4:**
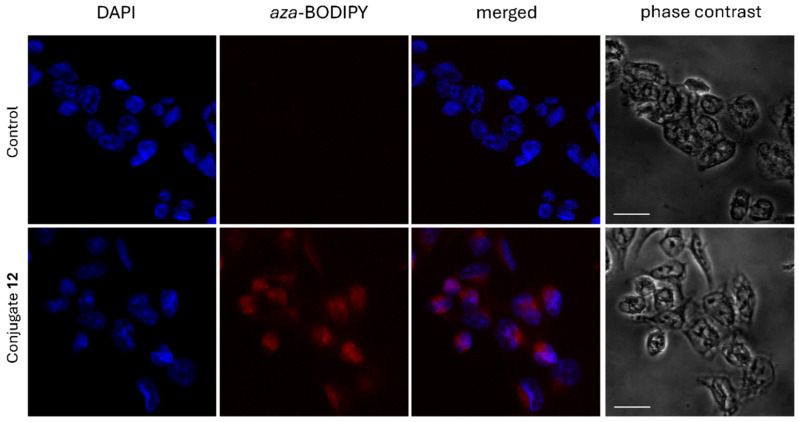
Confocal fluorescent microscopic images of T-47D cells incubated in the presence or absence of 10 µM *aza*-BODIPY–E2 conjugate **12**. Red fluorescent signal indicated cytosolic and to a lesser extent nuclear localization of the conjugate. Images were taken after 4 h treatments, nuclei were stained with DAPI. The scale bar represents 20 µm.

Fluorescent confocal microscopy experiments were performed on ER + T-47D cells to investigate the cellular localization of *aza*-BODIPY–E2 conjugate **12** (Figure 4). After 4 h treatment of T-47D cells with 10 µM conjugate **12**, the red fluorescence was observed both in the cytosol and within the cell nuclei. The results clearly indicate a massive uptake of the *aza*-BODIPY–E2 conjugate by T-47D cells. Furthermore, the nuclear entry of conjugate **12** could also be verified based on the red fluorescence observed not only in the cytosol but in the nuclei as well, albeit nuclear localization was of slightly less extent than cytosolic accumulation.

### 2.3. Photophysical Characterization of ***10*** and ***12***

**Figure 5:**
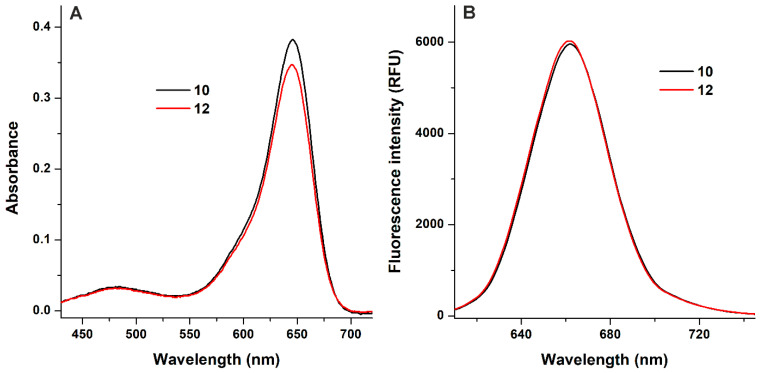
Representative absorption (**A**) and fluorescence emission (**B**) spectra of **10** and **12** (both 5 μM) in DMSO (see further details in Table 3).

Absorption and fluorescence emission spectra of **10** and **12** were recorded in DMSO (see the representative spectra in Figure 5). Both compounds showed absorption wavelength maxima at 645 nm, where **10** had slightly higher absorbance than **12**. Using 645 nm excitation wavelength, compounds **10** and **12** represented similar emission peaks with wavelength maxima at 664 nm and 663 nm, respectively (Figure 5). The absolute fluorescence quantum yields were approximately 18% (Table 3).

### 2.4. Docking Calculations

**Figure 6:**
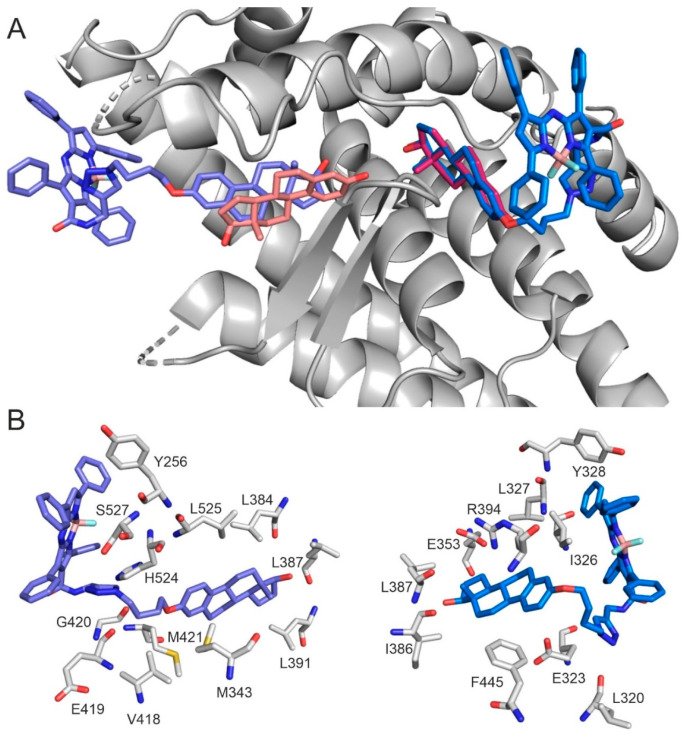
(**A**) Global view of *aza*-BODIPY conjugate (blue, marine) and E2 (salmon, magenta) binding in CBS (left) and ABS (right). E2 binding mode represents the experimental structure in CBS (Pdb code: 1a52 [35]) and the docked conformation in ABS [32]. (**B**) The close-up view of *aza*-BODIPY conjugate binding in CBS (left) and ABS (right). Target residues are represented as grey sticks.

Docking calculations on the apo conformation of human estrogen receptor alpha (hERα) were performed to investigate the binding mode of *aza*-BODIPY-labelled estradiol. The validity of the docking method was demonstrated in our previous studies [6,32]. In hERα, two binding sites were distinguished, classical and alternative (CBS, ABS), separated by a salt bridge of E353 and R394, respectively [32,33]. Separate docking searches were conducted, focusing on each binding site. The *aza*-BODIPY conjugate docked into both CBS and ABS via its estrogen moiety, and the ligand nicely overlapped with the natural reference E2 in ABS (Figure 6A).

CBS is a more closed and buried binding pocket from the bulk than ABS, and is suggested to mediate the classical genomic effect of E2 [32]. The interaction pattern of the docked derivative shows differences compared to E2. In the apo hERα, E2 interacts with E353 and R394 through its phenolic hydroxy group, which is essential for receptor activation [34]. This interaction is missing in the case of the *aza*-BODIPY conjugate. Furthermore, the steroid part of the labeled ligand shows a head-tail overlapping deviation from the natural agonist (Figure 6B).

ABS is more exposed to the bulk than CBS, resulting in higher accessibility for large ligands like the *aza*-BODIPY conjugate. In this pocket, the binding mode and pattern of the ligands’ steroid fragment are highly similar to those of E2 (Figure 6B).

## 3. Materials and Methods

### 3.1. Chemistry, General

Microwave-assisted syntheses were performed in the Anton-Paar Monowave 400 reactor. Melting points (Mp) were determined with a Kofler hot-stage apparatus and are uncorrected. Thin-layer chromatography was performed on silica gel 60 F254; (layer thickness 0.2 mm, (Merck)); eluent: a: 30% ethyl acetate/70% hexanes, b: 50% CH_2_Cl_2_/50% hexanes, c: 10% methanol/90% CH_2_Cl_2_, d: 5% methanol/95% CH_2_Cl_2_. The spots were detected with I_2_ or UV (365 nm) after spraying with 5% phosphomolybdic acid in 50% aqueous phosphoric acid and heating at 100–120 °C for 10 min. Flash chromatography was performed on silica gel 60, 40–63 μm (Merck, Rahway, NJ, USA). ^1^H NMR spectra were recorded in DMSO-d_6_ or CDCl_3_ solution with a Bruker DRX-500 instrument at 500 MHz. ^13^C NMR spectra were recorded with the same instrument at 125 MHz under the same conditions. Mass spectrometry: full scan mass spectra of the newly synthesized compounds were acquired in the range of 100 to 1100 *m*/*z* with a Q Exactive Plus quadrupole-orbitrap mass spectrometer (Thermo Fisher Scientific, Waltham, MA, USA) equipped with a heated electrospray (HESI). Analyses were performed in positive or negative ion mode by flow injection mass spectrometry with a mobile phase of 50% aqueous acetonitrile containing 0.1 *v*/*v*% formic acid (0.3 mL min^−1^ flow rate). Aliquots of 5 µL of samples were injected into the flow. The ESI capillary was adjusted to 3.5 kV and N_2_ was used as a nebulizer gas.

#### 3.1.1. Synthesis of Chalcone **5**

Benzaldehyde (**3**, 0.53 g, 5 mmol) was dissolved in a solution of sodium hydroxide (0.40 g, 10 mmol) in methanol (50 mL) and stirred at rt for 10 min. Acetophenone (**4**, 0.59 mL, 5 mmol) was added, and the stirring was continued at rt for 2 h. The solvent was removed under reduced pressure, and the residue was extracted with dichloromethane (3 × 10 mL). The combined organic layers were dried over sodium sulfate and the solvent was removed under reduced pressure. The crude compound **5** was obtained as a yellow solid (**5**: 0.86 g, 82%) and subjected to the next reaction step without purification.

#### 3.1.2. Synthesis of 4-Nitro-1,3-Diphenylbutan-1-One **6**

Chalcone **5** (0.50 g, 2.4 mmol) was dissolved in a solution of 0.2 equiv. of sodium hydroxide (19 mg, 0.48 mmol) in methanol (5 mL). This was followed by the addition of nitromethane (0.64 mL, 12 mmol) and the solution was stirred at 60 °C for 3 h. The solvent was removed under reduced pressure, and the residue was extracted with ethyl acetate (3 × 10 mL) and washed with brine (1 × 10 mL). The combined organic layers were dried over sodium sulfate, and the solvent was removed under reduced pressure. The residue was purified by flash chromatography with 15% ethyl acetate/85% hexanes as an eluent. Compound **6** was obtained as a pale yellow solid (0.52 g, 80%). **6**: Mp = 68.8–70.3 °C, Rf = 0.55^a^. Compound **6** was identical to the compound described in the literature [18].

#### 3.1.3. Synthesis of (3,5-Diphenyl-1H-Pyrrol-2-Yl)-(3,5-Diphenyl-Pyrrol-2-Ylidene)Amine **7**

##### Method A

The mixture of compound **6** (0.10 g, 0.38 mmol) and ammonium acetate (0.585 g, 7.6 mmol) was heated in a microwave reactor at 100 °C for 3 min. The reaction mixture was diluted with water and extracted with ethyl acetate (3 × 15 mL). The combined organic layers were dried over sodium sulfate and the solvent was removed under reduced pressure. The residue was purified by flash chromatography with 20% CH_2_Cl_2_/80% hexanes as an eluent. Compound **7** was obtained as a dark blue solid (40 mg, 48%). Mp = 286.0–288.2 °C, Rf = 0.65^b^. Compound **7** was identical to the compound described in the literature [18].

##### Method B

The mixture of compound **6** (0.10 g, 0.38 mmol), urea (0.46 g, 7.6 mmol), citric acid (0.22 g, 1.14 mmol), and water (5 mL) were heated in a microwave reactor at 150 °C for 30 min. The reaction mixture was then extracted with ethyl acetate (3 × 15 mL). The combined organic layers were dried over sodium sulfate and the solvent was removed under reduced pressure. The residue was purified by flash chromatography with 20% CH_2_Cl_2_/80% hexanes as an eluent. Compound **7** was obtained as a dark blue solid (43 mg, 51%). Mp = 286.0–288.2 °C, Rf = 0.65^b^. Compound **7** was identical to the compound described in the literature [18].

#### 3.1.4. Synthesis of *aza*-BODIPY **2**

A stirred solution of compound **7** (105 mg, 0.23 mmol) in toluene (3 mL) was treated with triethylamine (0.32 mL, 10 equiv.). The solution was stirred at rt for 15 min, then BF_3_·OEt_2_ (0.43 mL, 15 equiv.) was added dropwise and the solution was stirred under reflux for 5 h. After cooling to rt, the reaction was quenched using water (10 mL) and extracted with toluene (3 × 10 mL). The combined organic layers were dried over sodium sulfate and the solvent was removed under reduced pressure. The residue was purified by flash chromatography with 30% CH_2_Cl_2_/70% hexanes as an eluent. Compound **2** was obtained as a dark red solid (110 mg, 95%). Mp = 220.2–223.0 °C, Rf = 0.40^b^. Compound **2** was identical to the compound described in the literature [18,36]. ^1^H NMR (500 MHz, CDCl_3_) δ 7.05 (s, 2H), 7.42–7.50 (overlapping multiplets, 12H), 8.04–8.08 (overlapping multiplets, 8H).

#### 3.1.5. Formylation of Compound **2**

Next, 3 mL of phosphoryl chloride and 3 mL of DMF were stirred in a 25 mL round bottom flask for 5 min at 0 °C, and 30 min at rt. Then a solution of compound **2** (100 mg, 0.20 mmol) in 6 mL of 1,2-dichloroethane was added. The reaction mixture was heated to 70 °C and stirred for 24 h, and then the reaction was cooled to rt and poured slowly to a saturated solution of sodium bicarbonate at 0 °C and 1 h at rt. After extraction with CH_2_Cl_2_ (3 × 10 mL), the combined organic layers were dried over sodium sulfate and the solvent was removed under reduced pressure. The residue was purified by flash chromatography with CH_2_Cl_2_/hexane = 30/70 as an eluent. The crude product **8** (90 mg, 85%) was subjected to the next reaction step without purification.

#### 3.1.6. Oxidation of Compound **8**

Compound **8** (90 mg, 0.17 mmol) was dissolved in THF (45 mL) and H_2_O (15 mL), sulfamic acid (33 mg, 0.34 mmol) and sodium chlorite (31 mg, 0.34 mmol) were added and the reaction mixture was stirred at rt for 30 min. The mixture was diluted with ethyl acetate and poured onto a solution of sodium thiosulfate. The organic layer was extracted with ethyl acetate (3 × 20 mL), dried over anhydrous sodium sulfate, filtered, and the solvent was removed under reduced pressure. The product was purified by column chromatography with 3% methanol in CH_2_Cl_2_ as an eluent. Compound **9** was obtained as a dark blue solid (85 mg, 92%). Mp = 254.3–258.1 °C, Rf = 0.60^c^. ^1^H NMR (500 MHz, DMSO-d_6_) δ 7.49–7.63 (overlapping multiplets, 15H), 7.76–7.79 (overlapping multiplets, 3H), 8.11–8.18 (overlapping multiplets, 4H), 12.88 (s, OH). ^13^C NMR (125 MHz, DMSO-d_6_) δ 165.1 (COOH), 163.9 (1 × C), 154.5 (1 × C), 147.2 (1 × C), 145.8 (2 × C), 141.5 (1 × C), 140.5 (1 × C), 132.4 (1 × CH), 130.9 (1 × C), 130.7 (1 × C), 130.4 (2 × CH), 130.0 (1 × CH), 129.7 (1 × CH), 129.3 (2 × CH), 129.1 (2 × CH), 128.7 (4 × CH), 127.8 (2 × CH), 127.6 (2 × CH), 122.1 (1 × CH). ESI-HRMS: m/z: 542.18479 [M + H]^+^ (C_33_H_22_BF_2_N_3_O_2_ + H^+^ requires 542.18521 [M + H]^+^).

#### 3.1.7. Reaction of Compound **9** with Propargyl Amine

Compound **9** (81 mg, 0.15 mmol) was dissolved in CH_2_Cl_2_ (3 mL), EDC (35 mg, 0.225 mmol) and HOBt (101 mg, 0.75 mmol) were added, and the reaction mixture was stirred at rt for 10 min. Then propargylamine (33 mg, 0.60 mmol) was added, and the mixture was stirred at rt for 4 h. The solvent was evaporated under reduced pressure, and the crude product was subjected to column chromatograpy with CH_2_Cl_2_ as an eluent. Compound **10** was obtained as a dark blue solid (80 mg, 92%). Mp = 260.2–263.4 °C, Rf = 0.90^d^. ^1^H NMR (500 MHz, CDCl_3_) δ 2.09 (s, 1H, C≡CH), 3.96 (m, 2H, NCH_2_), 5.53 (t, *J* = 5.0 Hz, NH), 7.14 (s, 1H), 7.43–7.51 (overlapping multiplets, 12H), 7.77–7.83 (overlapping multiplets, 4H), 8.03–8.07 (overlapping multiplets, 4H). ^13^C NMR (125 MHz, CDCl_3_) δ 164.1 (CONH), 163.8 (1 × C), 155.2 (1 × C), 147.9 (1 × C), 146.5 (2 × C), 142.7 (1 × C), 141.0 (1 × C), 132.0 (1 × CH), 131.5 (1 × C), 131.2 (1 × C), 130.9 (2 × CH), 130.7 (1 × C), 130.4 (1 × C), 130.3 (1 × CH), 130.2 (1 × CH), 129.9 (1 × CH), 129.8 (1 × CH), 129.5 (3 × CH), 128.8 (2 × CH), 78.8 and 71.8 (C≡CH), 29.5 (NCH_2_). ESI-HRMS: m/z: 579.21708 [M + H]^+^ (C_36_H_25_BF_2_N_4_O + H^+^ requires 579.21685 [M + H]^+^).

#### 3.1.8. Synthesis of *aza*-BODIPY–E2 Conjugate **12**

To a stirred solution of compound **10** (70 mg, 0.12 mmol) in dry toluene (3 mL), SPhos (10 mg, 0.024 mmol) and CuI (2.3 mg, 0.012 mmol) were added. The solution was treated with estradiol azide **11** (89 mg, 0.24 mmol) and DIPEA (0.17 mL, 0.96 mmol). The solution was stirred under reflux for 2 h. After cooling to rt, the solvent was removed under reduced pressure and the residue was purified by flash chromatography with methanol/CH_2_Cl_2_ = 2/8 as an eluent. Compound **12** was obtained as a dark blue solid (102 mg, 90%). Mp = 205.9–208.0 °C, Rf = 0.45^d^. ^1^H NMR (500 MHz, CDCl_3_) δ 0.77 (s, 3H, 18-H_3_), 2.82–2.85 (m, 2H, 6-H_2_), 3.71 (t, 1H, *J* = 8.4 Hz, 17-H), 3.93 (m, 2H) and 4.36–4.45 (overlapping multiplets, 4H): OCH_2_ and 2 × NCH_2_, 6.05 (s, 1H, NH), 6.60 (s, 1H, 4-H), 6.67 (d, 1H, *J* = 8.5 Hz, 2-H), 7.12 (s, 1H), 7.18 (d, 1H, *J* = 8.5 Hz, 1-H), 7.39–7.52 (overlapping multiplets, 13H), 7.75–7.77 (overlapping multiplets, 4H), 8.02–8.06 (overlapping multiplets, 4H). ^13^C NMR (125 MHz, CDCl_3_) δ 164.6 (CONH), 163.7 (1 × C), 156.6 (C-3), 154.8 (1 × C), 147.6 (1 × C), 146.3 (2 × C),142.7 (1 × C), 140.7 (1 × C), 138.1 (1 × C), 133.0 (1 × C), 131.9 (1 × CH), 131.5 (1 × C), 131.0 (1 × C), 130.8 (2 × CH), 130.7 (1 × C), 130.5 (1 × C), 130.2 (1 × CH), 130.1 (1 × CH), 129.9 (2 × CH), 129.8 (2 × CH),129.4 (3 × CH), 128.8 (2 × CH), 128.7 (2 × CH), 128.2 (2 × CH), 128.0 (2 × CH), 127.0 (1 × C), 126.4 (1 × CH), 120.4 (1 × CH), 114.4 (1 × CH), 111.9 (1 × CH), 81.8 (C-17), 66.7 (OCH_2_), 50.2 (1 × CH_2_), 50.0 (1xCH), 43.9 (1 × CH), 43.3 (C-13), 38.8 (1 × CH), 36.6 (1 × CH_2_), 35.1 (1 × CH_2_), 30.6 (1 × CH_2_), 29.8 (1 × CH_2_), 27.2 (1 × CH_2_), 26.3 (2 × CH_2_), 23.0 (1 × CH_2_), 10.94 (13-CH_3_). ESI–HRMS: m/z: 948.45606 [M + H]^+^ (C_58_H_56_BF_2_N_7_O_3_ + H^+^ requires 948.45848 [M + H]^+^).

### 3.2. UV-Vis and Fluorescence Spectroscopic Measurements

Stock solutions (5000 μM) of **10** and **12** were prepared in dimethyl sulfoxide (DMSO; spectroscopic grade; Merck, Darmstadt, Germany). Absorption and fluorescence emission spectra were collected in DMSO at 25 °C, using a U-3900 UV-Vis spectrophotometer and a F-4500 fluorescence spectrophotometer (both Hitachi, Tokyo, Japan), respectively.

The absolute fluorescence quantum yields (Φ_FL_) of **10** and **12** were determined in DMSO as previously described [37], applying a fluorescence spectrofluorometer (HORIBA Jobin-Yvon, NanoLog FL3-2iHR, Paris, France) with an integrating sphere accessory (HORIBA Quanta-phi F-3029), and the NanoLog’s QY/colorimetry software: FluorEssence™ (version number: 3.9.0.1; Origin version: 8.6001) for the calculations.

### 3.3. Cell Cultures

For the pharmacological experiments, we have used a set of human breast carcinoma cell lines, briefly, the estrogen-receptor positive T47-D, the estrogen-receptor-negative MDA-MB-231, and luciferase transfected T47D-KBluc. NIH/3T3 mouse embryo fibroblasts were utilized as non-cancerous control. Cells were purchased from ECACC (European Collection of Cell Cultures, Salsbury, UK), except for the T47D-KBluc (American Tissue Culture Collection, Manassas, VA, USA). T47D-KBluc cells were cultivated in RPMI-1640, while all the other cell lines were grown in Eagle’s Minimum Essential Medium (EMEM, Capricorn Scientific GmbH, Ebsdorfergrund, Germany) supplemented with 10% heat-inactivated fetal bovine serum (FBS), 1% non-essential amino acids (NEAA), and 1% antibiotic-antimycotic mixture (penicillin-streptomycin). The cells were maintained at 37 °C in a humidified atmosphere containing 5% CO_2_.

### 3.4. MTT Assay

MTT assay is a standard method to examine the compound’s direct effect on the cell number of ER-positive and negative cell lines. During the MTT assay, cells were seeded into 96-well plates at a density of 5000, 10,000, and 40,000 cells/well for 24 h, 72 h incubation, and for the estrogen activity test, respectively. Twenty-four hours after the seeding, the cells were treated with a set of concentrations of the test compound (0.1 to 30 µM). After the appropriate incubation time, 5 mg/mL MTT solution (3-(4,5-dimethyl-azole-2-yl)-2,5-diphenyl-2H-tetrazolium bromide) was added to each well. After 4 h, the supernatant was removed, and the formazan crystals were dissolved in DMSO. The absorbance was measured with a microplate reader at 545 nm (BMG Labtech, Ortenberg, Germany). Wells with untreated cells were utilized as control, and inhibition values were calculated by GraphPad Prism v 9.0 software (GraphPad Software, San Diego, CA, USA).

### 3.5. Luciferase Reporter Gene Assay

The transfected cell line T47-D-KBluc was used to prove the estrogenic activity of the new labeled compound. Precisely, cells were seeded at the density of 40,000 cells/well in RPMI-1640 without phenol red and left to grow until 90% confluency, and then the media was changed into the same media containing charcoal-stripped FBS. Phenol red deprivation and charcoal-stripped FBS are required to eliminate all estrogen from the system. After two days of adaptation, cells were treated with the test compound, E2, and fulvestrant as a reference compound for estrogen agonist and antagonist effect, respectively. After 24 h of incubation under cell culturing conditions, the OneGlo Luciferase assay kit was used according to the manufacturer’s recommendations. Briefly, the luciferase substrate was dissolved in the Assay Buffer. After aspiration of the supernatant from the cells, the previously prepared reaction buffer was added to each well, and the cells were lyzed. After 5 min of incubation, the luminescence signal was measured by a microplate reader, and the signals were collected for 5 s/well. (BMG Optima, BMG Labtech, Ortenberg, Germany). Cell-free wells were used as blank; untreated control was used to determine the baseline and to check for any estrogenic activity in the assay system except the tested compounds. E2 (40 pM) was used as a reference, and fulvestrant combined with E2 was used to check the antiestrogenic effect.

### 3.6. Fluorescent Confocal Microscopy

To investigate the intracellular localization of *aza*-BODIPY–E2 conjugate **12**, T-47D cells were seeded into 24-well plates containing glass coverslips in each well in 1 × 10^5^ cells/well density. On the following day, cells were treated with 10 µM of conjugate 12 in complete culture medium for 4 h. Then the conjugate **12** containing medium was removed, the cells were washed twice with PBS and fixed by ice-cold methanol:acetone (1:1) for 10 min. Cell nuclei were stained with 4′,6-diamidino-2-phenylindole (DAPI; 1 µg/mL) for 10 min. Finally, the coverslips were mounted on glass slides using Fluoromount mounting medium (Merck, Darmstadt, Germany) to ensure sample stability and fluorescence preservation. Fluorescent signals were observed and images were taken using an Olympus Fluoview Fv10i confocal microscope with a 60× objective. The samples were excited at 405 nm for DAPI (40% laser intensity), and 635 nm for *aza*-BODIPY–E2 conjugate 12 (40% laser intensity) with emissions at 461 nm for DAPI and 668 nm for BODIPY. Images were processed by Olympus Fluoview ver3.1a Viewer.

### 3.7. Computational Simulations

*Preparation of ligands*. *aza*-BODIPY-labelled ligand was built in Maestro [38]. Raw structures were minimized using MOPAC [39], a semiempirical quantum chemistry program package with PM7 parametrization. The gradient norm was set to 0.001. Force calculations with positive force constant matrices were applied on the energy-minimized structures. These optimized ligand structures were used for docking calculations.

*Preparation of target.* The structure of the ligand-free (apo) human estrogen receptor alpha ligand binding domain (hERα LBD) was obtained from the Protein Data Bank [40] (PDB code: 2b23; [41]). Water molecules, ions, and coactivator peptide fragments as well as the B conformation of alternative residues were cut from the original experimental structure. The modified CME residues were mutated to cysteine according to the natural sequence of hERα LBD. The N- and C-terminals of the protein were capped by adding acetyl (ACE) and imino-methyl (NME) groups using PyMol [42], respectively.

*Docking calculations.* Docking calculations were performed with Autodock 4.2.6 [43] using the minimized and equilibrated ligand structures on target’s region including either the classical or the alternative binding site (CBS, ABS) determined in previous study [32]. The grid box for each calculation was generated by Autogrid 4.2.6. [43], with 120 gridpoints along all axes spacing of 0.375 Å, centered to cover either CBS or ABS, and bulk to have even the longest ligand enough place to find the best docking pose. Gasteiger–Marsili partial charges to both the ligand and the receptor atoms were assigned by AutodockTools [43] using a united-atom representation for non-polar moieties. The receptor was treated rigidly; however, flexibility was allowed at all active torsions of the ligands. Lamarckian genetic algorithm was used for global searching. After 100 docking runs, ligand conformations were ranked by the corresponding calculated interaction energy values and subsequently clustered using a tolerance of 3.5 Å root mean square deviation (RMSD) between cluster members. The rank 1 was analyzed and selected as representative structure for each ligand. Parameters of carbon atoms were applied for boron in docking calculations for all ligands.

## 4. Conclusions

A facile, fluorescent labeling methodology has been elaborated for the development of a novel red-emitting E2 conjugate. The *aza*-BODIPY dye was attached to the steroid at the phenolic hydroxy function via a linker with a four-carbon unit and a triazole coupling moiety. For the first time in the literature, we have carried out the selective conjugation of E2 at the A-ring. The potent estrogenic activity of the newly synthesized fluorescent conjugate was proved via transcriptional luciferase assay. Despite attaching a much larger and structurally different molecular moiety to E2 than recently [6], the resulting derivative retained its estrogenic activity. The cellular localization of the conjugate was investigated by fluorescent confocal microscopy on ER+ T-47D cells. The red fluorescence was observed not only in the cytosol but in the nuclei as well. Docking experiments uncovered the binding mode of the conjugate in both ABS and CBS. The *aza*-BODIPY conjugate docked into both CBS and ABS via its estrogen moiety, and the ligand nicely overlapped with the natural reference E2 in ABS. However, the steroid part of the labeled ligand showed a head-tail overlapping deviation from the natural agonist in CBS. Due to its substantial estrogenic activity and favorable optical characteristics, the newly synthesized *aza*-BODIPY-E2 conjugate might find its utilization in several biomedical fields. The present red-emitting *aza*-BODIPY–E2 conjugate complements the chemical space of the green-emitting derivatives published by us recently [6], as labeled estrogens operating in different wavelength ranges may be suitable for different applications. The fluorescent conjugate emitting at higher wavelengths, due to its deeper tissue penetration, may serve as a foundation for live-cell imaging techniques, to provide images with high spatial and temporal resolution.

## Figures and Tables

**Table 1:** Effect of the reaction conditions on the dimerization of compound **6** under microwave irradiation.

Entry	Reagent (Equiv.)	Acid (Equiv.)	Solvent	Temp (°C)	Time (min)	6 ^a^ (%)
1	NH_4_OAc (20)	-	ethanol	100	30	39
2	NH_4_OAc (20)	-	ethanol	150	30	33
3	NH_4_OAc (20)	-	water	100	30	18
4	NH_4_OAc (20)	-	water	150	30	20
5	NH_4_OAc (20)	-	-	100	3	48
6	NH_4_OAc (20)	-	-	150	3	8
7	urea (20)	-	ethanol	100	30	-
8	urea (20)	-	ethanol	150	30	-
9	urea (20)	AA (10)	ethanol	100	30	30
10	urea (20)	AA (10)	ethanol	150	30	40
11	urea (20)	AA (10)	water	100	30	32
12	urea (20)	AA (10)	water	150	30	42
13	urea (20)	CA (3)	ethanol	100	30	35
14	urea (20)	CA (3)	ethanol	150	30	40
15	urea (20)	CA (3)	water	100	30	38
16	urea (20)	CA (3)	water	150	30	51

^a^ Flash chromatography yield.

**Table 2:** The antiproliferative effect of conjugate **12** in the panel of breast cancer cells with different receptor status. Growth inhibitions are given in % after 10 and 30 µM treatment ± SEM.

Cell Type	Receptor Status	Concentration (µM)	Inhibition (%)	SEM
MDA-MB-231	ER–, PR–, HER2–	10	53.30	3.17
		30	57.38	2.59
T-47-D	ER+, PR+, HER2–	10	−4.30	3.46
		30	20.72	2.23
NIH/3T3		10	27.37	0.87
		30	15.40	1.70

**Table 3:** UV-Vis and fluorescence spectroscopic properties of **10** and **12** in DMSO, including absorption wavelength maximum (λ_abs_), fluorescence emission wavelength maximum (λ_em_), stokes shift (Δλ), maximum molar absorptivity (ε_max_), and fluorescence quantum yield (Φ_FL_).

Compound	Solvent	λ_abs_ (nm)	λ_em_ (nm)	Δλ (nm)	ε_max_ (M^−1^ cm^−1^)	Φ_FL_ (%)
**10**	DMSO	645	664	19	7.40 × 10^4^	18.1
**12**	DMSO	645	663	18	6.74 × 10^4^	18.2

## Data Availability

Data are available upon request.

## Supplementary Materials

The following supporting information can be downloaded at: https://www.mdpi.com/article/10.3390/ijms26157075/s1.
