# Supplementary material for: The Red Shift in Estrogen Research: An Estrogen-Receptor Targeted aza-BODIPY–Estradiol Fluorescent Conjugate
Source: Int J Mol Sci. 2025 Jul 23;26(15):7075. doi: 10.3390/ijms26157075 (PMC12346466; doi:10.3390/ijms26157075)
Supplement: Supplementary file 1 [file ijms-26-07075-s001.zip › ijms-3691377-supplementary.pdf]

The Red Shift in Estrogen Research: An Estrogen-Receptor Targeted aza-BODIPY–Estradiol Fluorescent Conjugate

Tamás Hlogyik,[1,#] Noémi Bózsity,[2,#] Rita Börzsei,[3,*] Benjámin Kovács,[1] Péter Labos,[2] Csaba Hetényi,[3] Mónika Kiricsi,[4] Ildikó Huliák,[4] Zoltán Kele,[5] Miklós Poór,[6,7] János Erostyák,[8,9] Attila Hunyadi,[1,10] István Zupkó,[2] Erzsébet Mernyák[1*]

[1] Institute of Pharmacognosy, University of Szeged, Eötvös u. 6, H-6720 Szeged, Hungary

[2] Institute of Pharmacodynamics and Biopharmacy, University of Szeged, Eötvös u. 6, H-6720 Szeged, Hungary

[3] Department of Pharmacology and Pharmacotherapy, Medical School, University of Pécs, Szigeti út 12, H-7624 Pécs, Hungary

[4] Department of Biochemistry and Molecular Biology, University of Szeged, Közép fasor 52, H-6726 Szeged, Hungary

[5] Department of Medicinal Chemistry, University of Szeged, Dóm tér 8, H-6720 Szeged, Hungary

[6] Department of Laboratory Medicine, Medical School, University of Pécs, Ifjúság útja 13, Pécs

[7] Molecular Medicine Research Group, János Szentágothai Research Centre, University of Pécs, Ifjúság útja 20, Pécs H-7624, Hungary

[8] Department of Experimental Physics, Faculty of Sciences, University of Pécs, Ifjúság útja 6, Pécs H-7624, Hungary; erostyak@fizika.ttk.pte.hu

[9] Molecular Biophysics Research Group, János Szentágothai Research Centre, University of Pécs, Ifjúság útja 20, Pécs H-7624, Hungary; erostyak@fizika.ttk.pte.hu

[10] HUN-REN-SZTE Biologically Active Natural Products Research Group, Eötvös u. 6, H-6720 Szeged, Hungary

*Corresponding author: rita.borzsei@gmail.com; mernyak.erzsebet@szte.hu

#These authors contributed equally to this work.

^{1}H and ^{13}C NMR spectra of the newly synthesized compounds

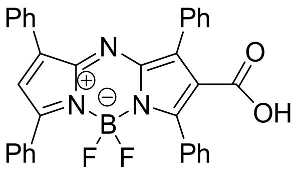

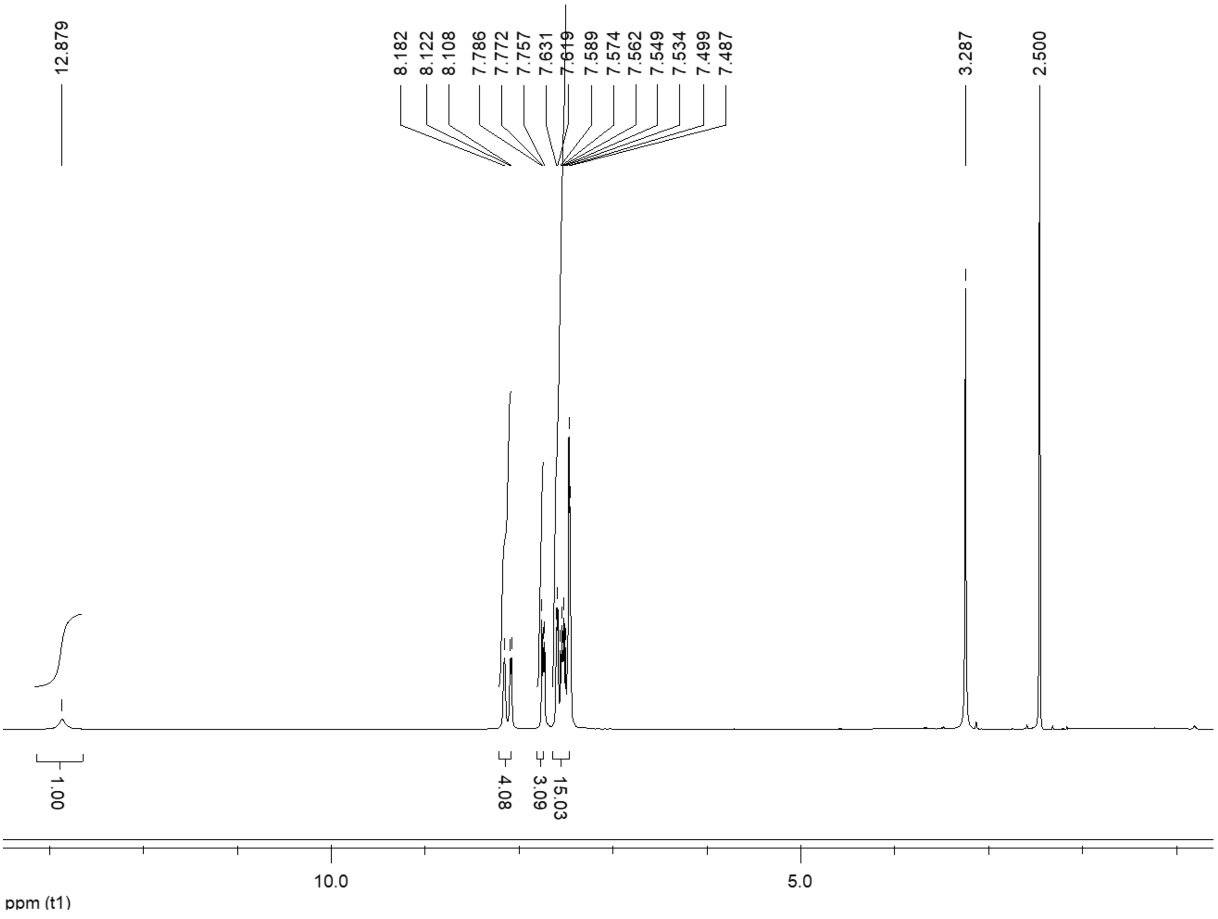

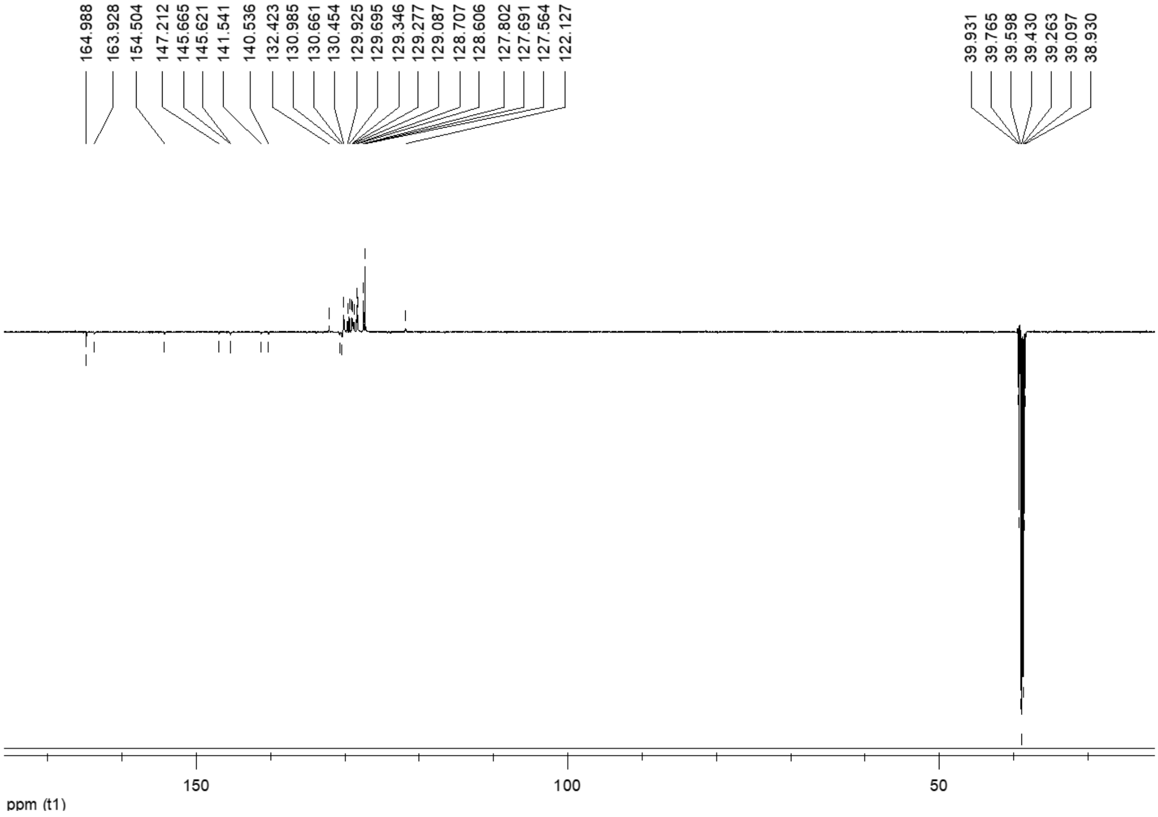
164.988
163.928
154.504
147.212
145.665
145.621
141.541
140.536
132.423
130.985
130.661
130.454
129.925
129.695
129.346
129.277
129.087
128.707
128.606
127.802
127.691
127.564
122.127
39.931
39.765
39.598
39.430
39.263
39.097
38.930
150
100
50
ppm (t1)

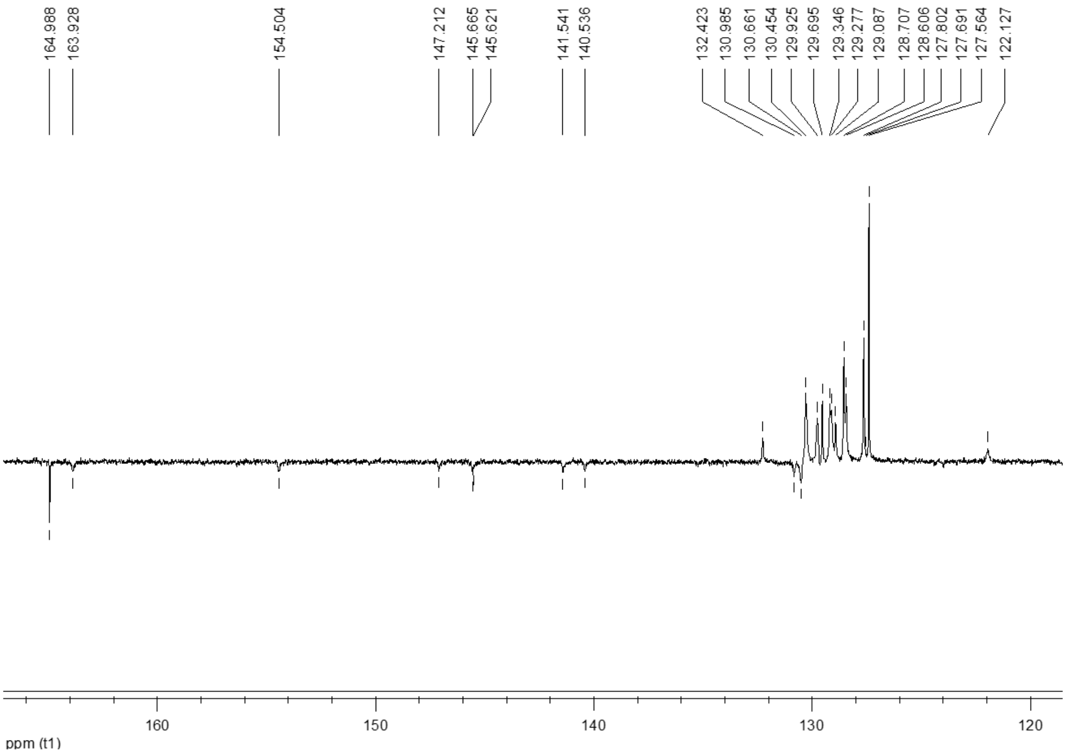
164.988
163.928
154.504
147.212
145.665
145.621
141.541
140.536
132.423
130.985
130.661
130.454
129.925
129.695
129.346
129.277
129.087
128.707
128.606
127.802
127.691
127.564
122.127
160
150
140
130
120
ppm (t1)

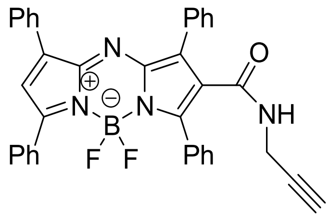
Ph
Ph
N
O
N
B
N
NH
Ph
F
F
Ph

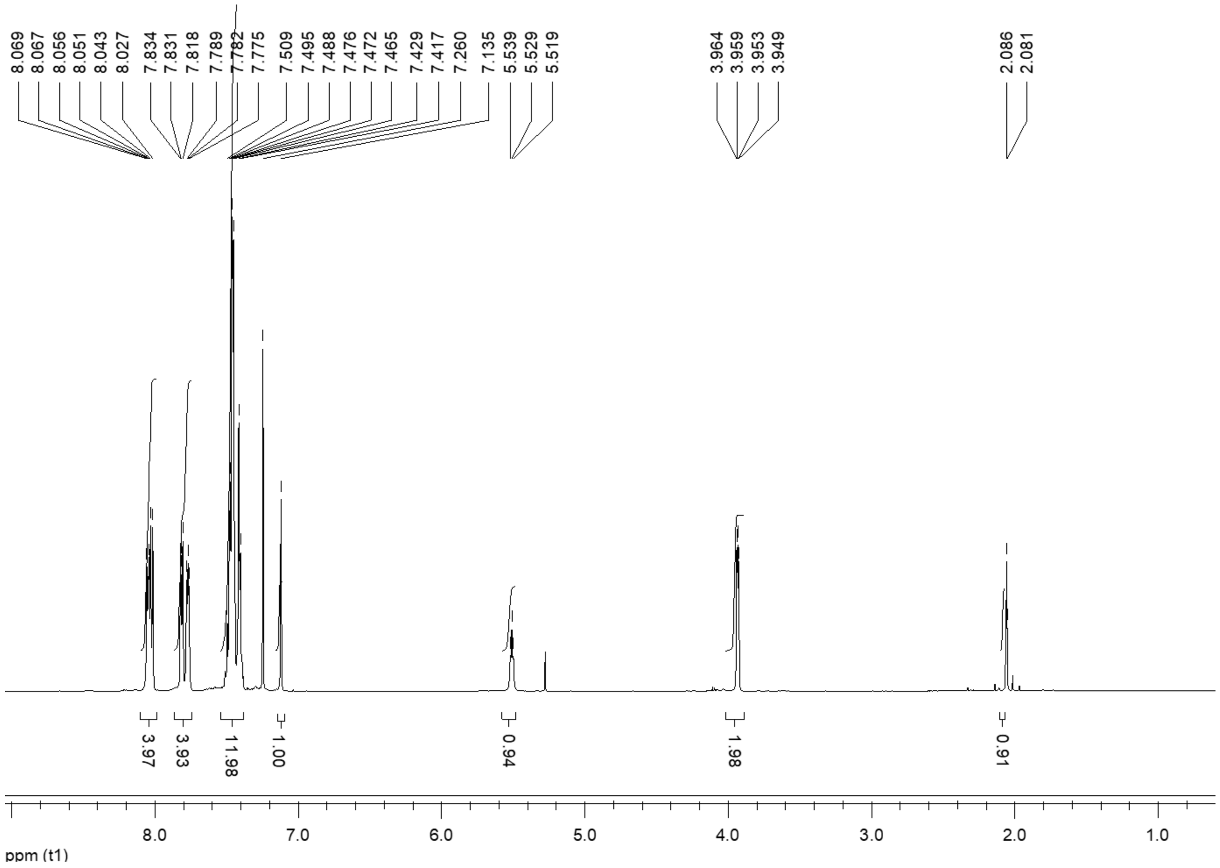
8.069
8.067
8.056
8.051
8.043
8.027
7.834
7.831
7.818
7.789
7.782
7.775
7.509
7.495
7.488
7.476
7.472
7.465
7.429
7.417
7.260
7.135
5.539
5.529
5.519
3.964
3.959
3.953
3.949
2.086
2.081
3.97
3.93
11.98
1.00
0.94
1.98
0.91
8.0
7.0
6.0
5.0
4.0
3.0
2.0
1.0
ppm (t1)

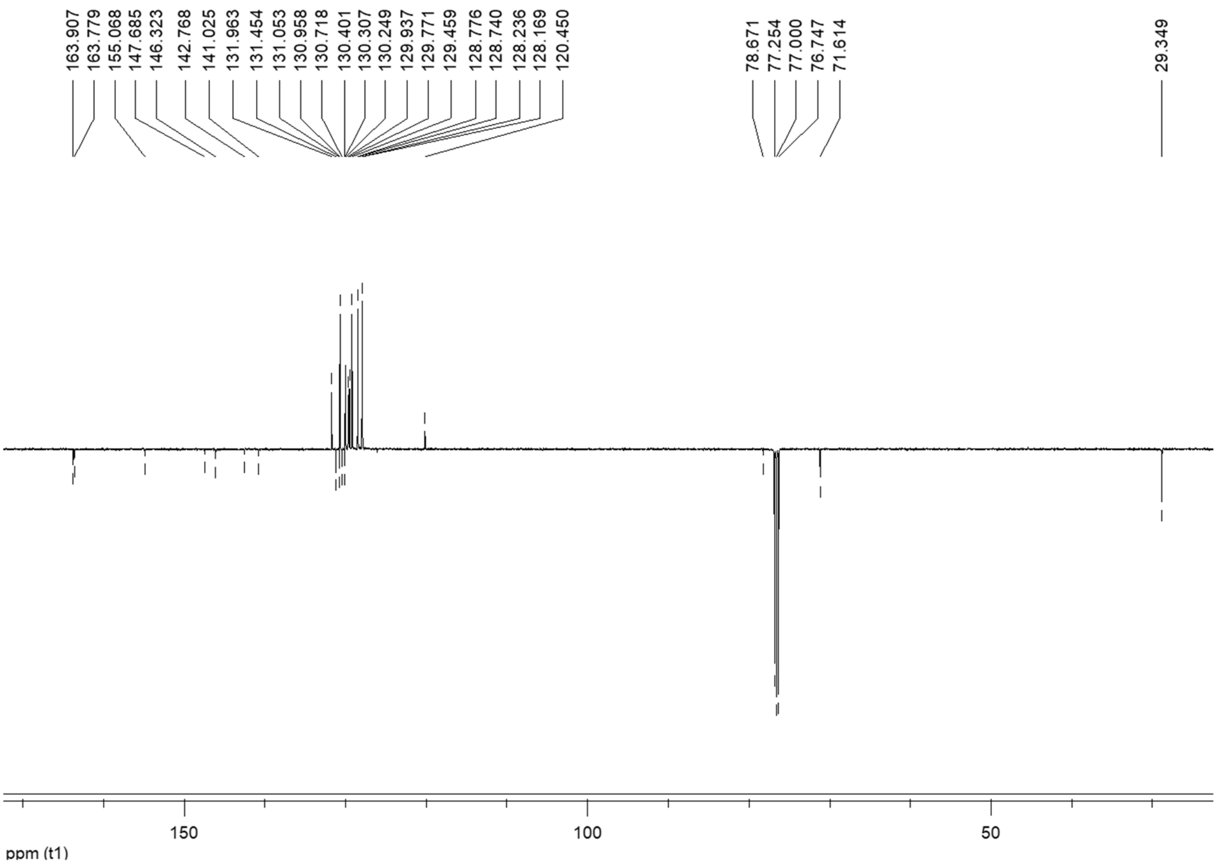
163.907
163.779
155.068
147.685
146.323
142.768
141.025
131.963
131.454
131.053
130.958
130.718
130.401
130.307
130.249
129.937
129.771
129.459
128.776
128.740
128.236
128.169
120.450
78.671
77.254
77.000
76.747
71.614
29.349
150
100
50
ppm (t1)

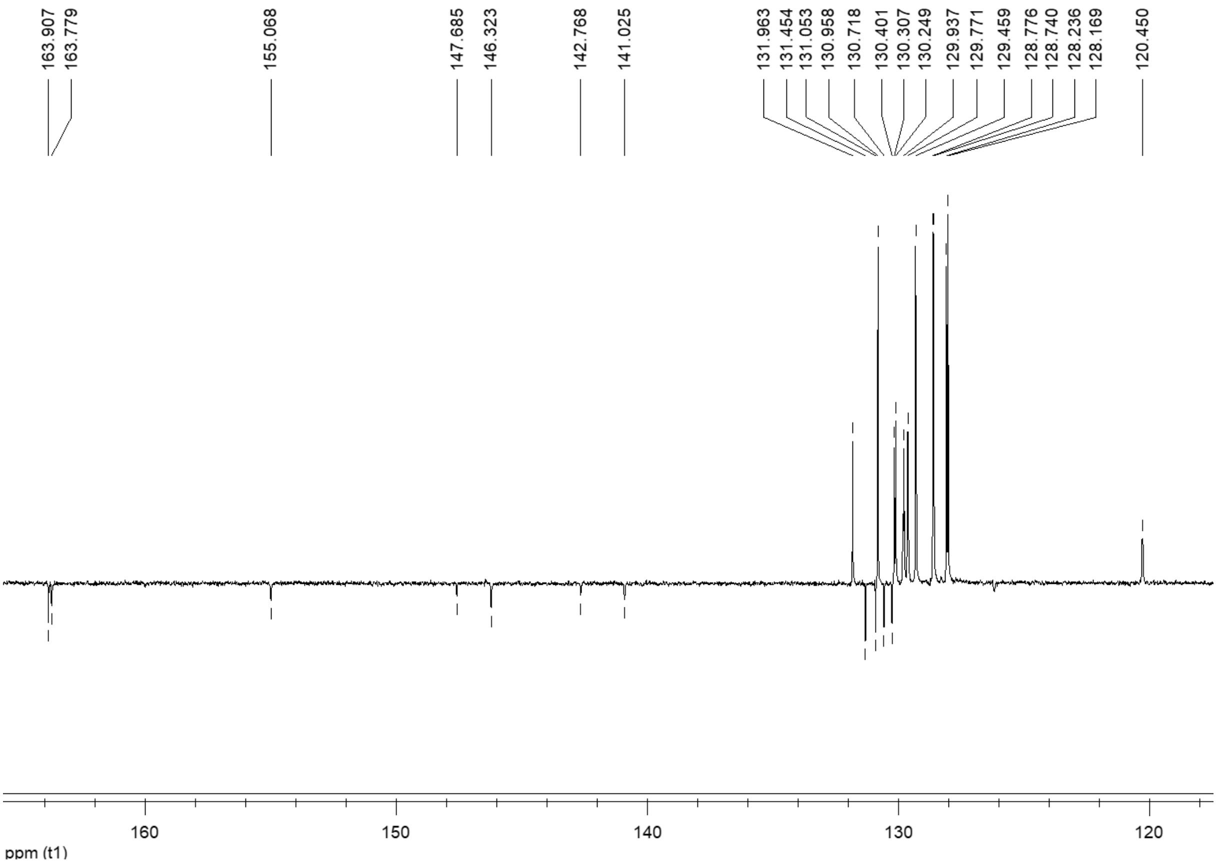
163.907
163.779
155.068
147.685
146.323
142.768
141.025
131.963
131.454
131.053
130.958
130.718
130.401
130.307
130.249
129.937
129.771
129.459
128.776
128.740
128.236
128.169
120.450
160
150
140
130
120
ppm (t1)

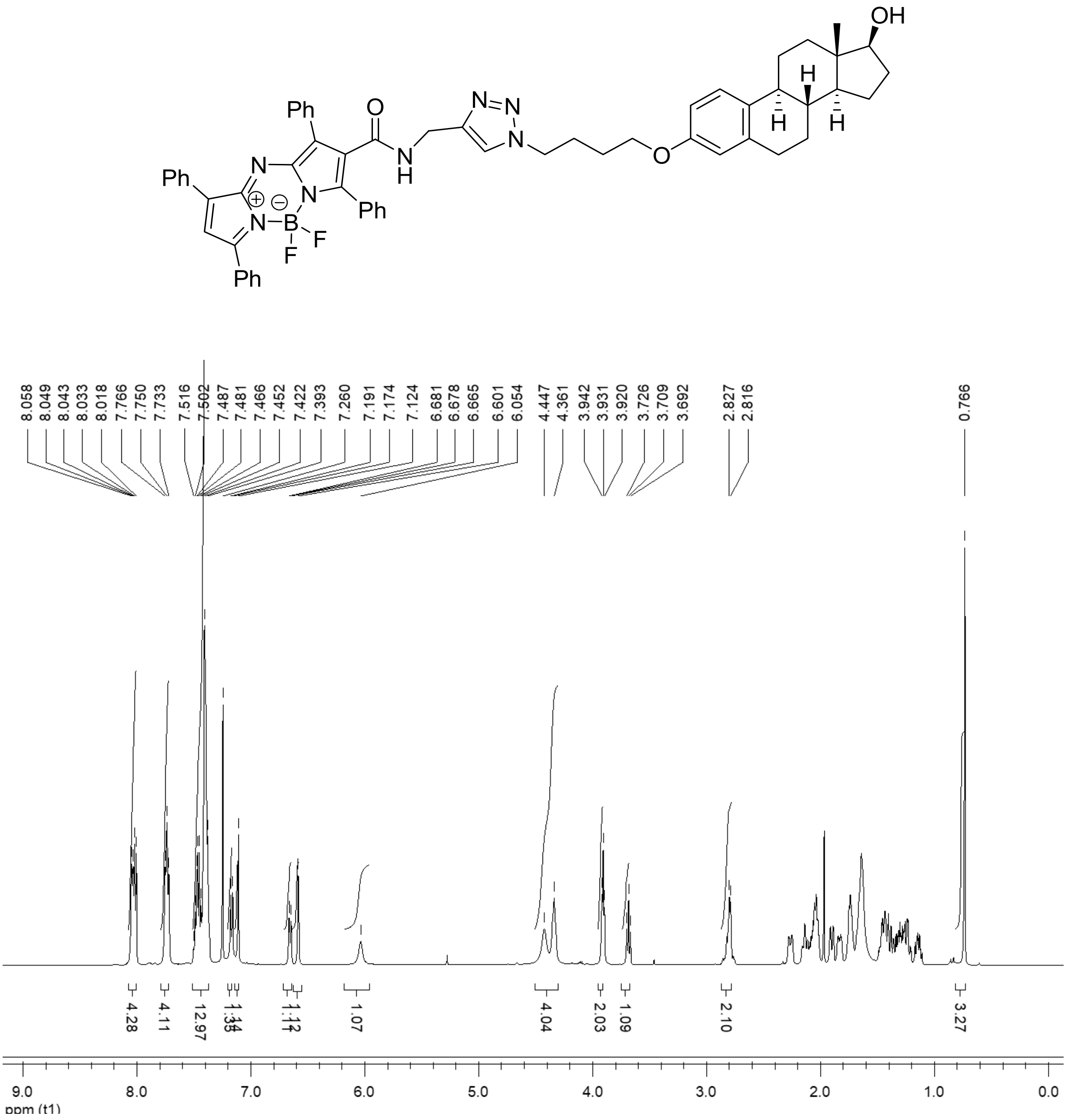

Ph
O
N=N
OH
H
N
NH
N
Ph
Ph
N
B
F
F
O
Ph
8.058
8.049
8.043
8.033
8.018
7.766
7.750
7.733
7.516
7.502
7.487
7.481
7.466
7.452
7.422
7.393
7.260
7.191
7.174
7.124
6.681
6.678
6.665
6.601
6.054
4.447
4.361
3.942
3.931
3.920
3.726
3.709
3.692
2.827
2.816
0.766
4.28
4.11
12.97
1.07
4.04
2.03
1.09
2.10
3.27
9.0
8.0
7.0
6.0
5.0
4.0
3.0
2.0
1.0
0.0
ppm (t1)

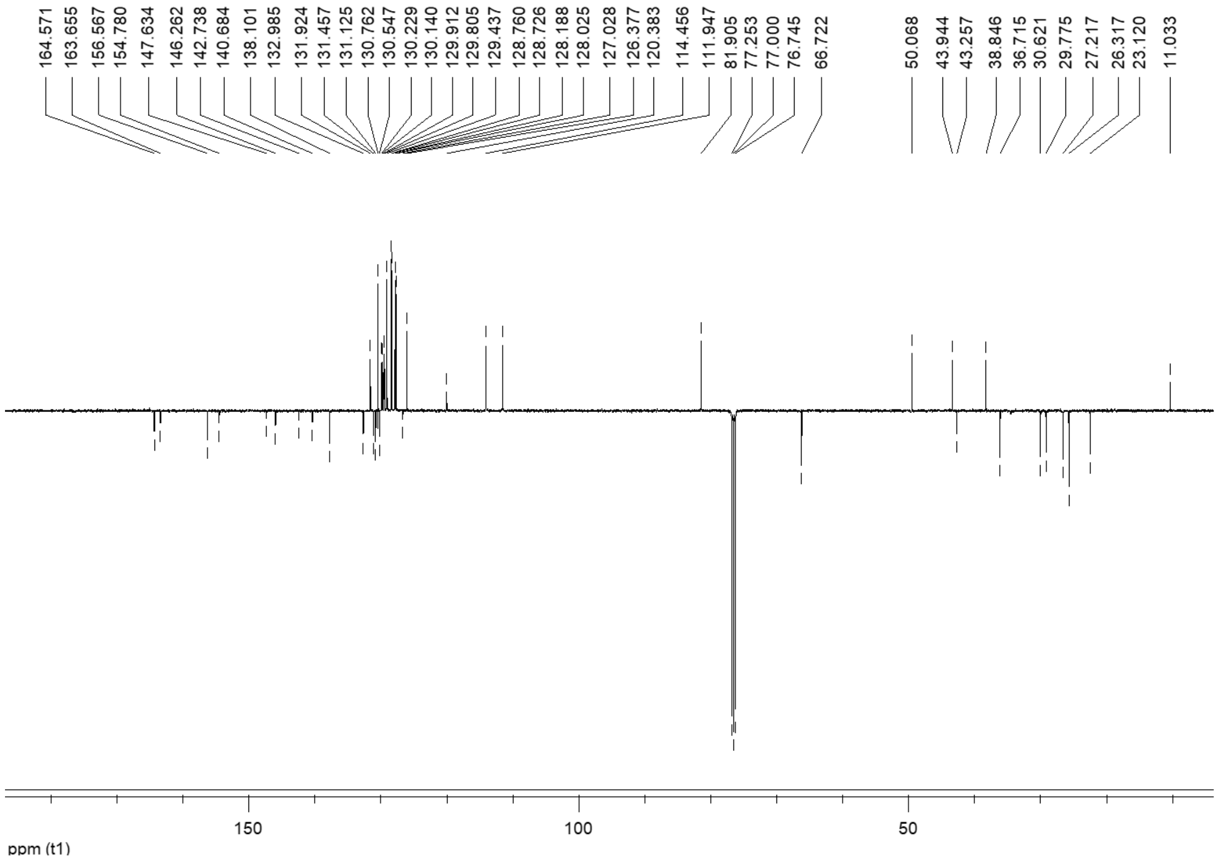
164.571
163.655
156.567
154.780
147.634
146.262
142.738
140.684
138.101
132.985
131.924
131.457
131.125
130.762
130.547
130.229
130.140
129.912
129.805
129.437
128.760
128.726
128.188
128.025
127.028
126.377
120.383
114.456
111.947
81.905
77.253
77.000
76.745
66.722
50.068
43.944
43.257
38.846
36.715
30.621
29.775
27.217
26.317
23.120
11.033
150
100
50
ppm (t1)

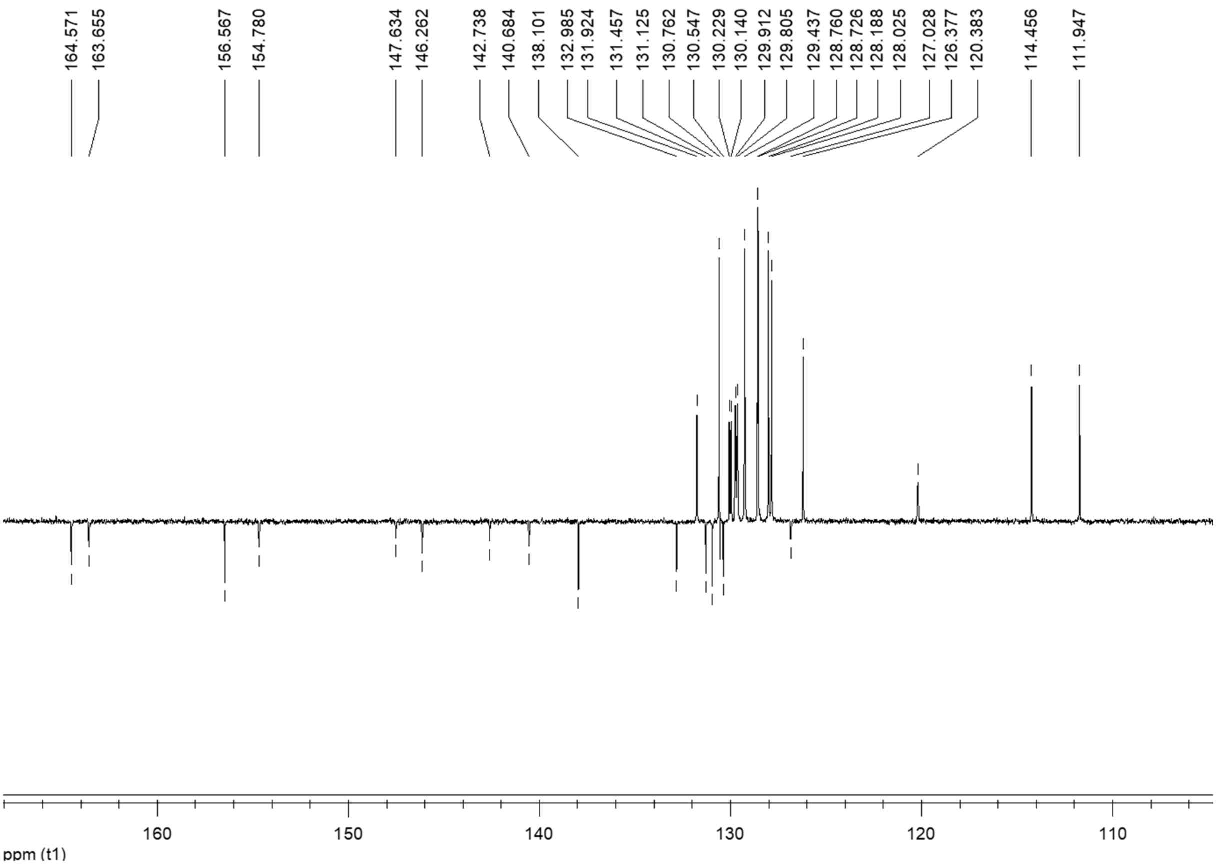
164.571
163.655
156.567
154.780
147.634
146.262
142.738
140.684
138.101
132.985
131.924
131.457
131.125
130.762
130.547
130.229
130.140
129.912
129.805
129.437
128.760
128.726
128.188
128.025
127.028
126.377
120.383
114.456
111.947
160
150
140
130
120
110
ppm (t1)
